# Detection Rates of Hepatitis B Surface and Core-related Antigens Using Novel Highly Sensitive Assays in Chronic Hepatitis B Patients With Hepatitis B Surface Antigen Seroclearance

**DOI:** 10.1016/j.gastha.2024.06.013

**Published:** 2024-07-03

**Authors:** Taiki Okumura, Satoru Joshita, Yoshiyuki Kitamura, Haruka Sagi, Takeji Umemura

**Affiliations:** 1 Division of Gastroenterology and Hepatology, Department of Medicine, Shinshu University School of Medicine, Matsumoto, Japan; 2 Department of Health Promotion Medicine, Shinshu University School of Medicine, Matsumoto, Japan; 3 Department of Internal Medicine, NHI Yodakubo Hospital, Nagawa, Japan; 4 Research and Development Division, Fujirebio Inc., Hachioji, Japan; 5 Department of Advanced Therapeutic Endoscopy, Shinshu University School of Medicine, Matsumoto, Japan; 6 Consultation Center for Liver Diseases, Shinshu University Hospital, Matsumoto, Japan

Hepatitis B virus (HBV) infection is a global health problem that causes morbidity and mortality in afflicted patients. The World Health Organization estimated 296 million people living with chronic hepatitis B infection in 2019, leading to approximately 820,000 deaths, mostly from cirrhosis and hepatocellular carcinoma (HCC).^1^ HBV infection outcomes vary from spontaneous clearance to viral persistence, the latter of which may progress to liver cirrhosis and HCC.^2^^,^^3^ As such, serological biomarkers are needed to accurately evaluate disease status. Regarding biomarkers associated with HBV infection, hepatitis B surface antigen (HBsAg), HBV DNA, and hepatitis B core-related antigen (HBcrAg) levels have all been used in the clinical setting to estimate HBV replication activity.

HBsAg seroclearance is the primary goal of HBV treatment, with patients achieving such a status generally showing a good prognosis. However, HBV cannot be completely eradicated due to the persistence of intrahepatic covalently closed circular DNA and integrated HBV DNA in hepatocytes. Thus, even patients with HBsAg seroclearance harbor the risk of HCC.^4^

Two novel high-sensitivity assays based on "immunoassay for total antigen including complex via pretreatment (iTACT)" technology have recently been developed for HBsAg and HBcrAg monitoring.^5^^,^^6^ The iTACT assay involves sample pretreatment with acids and/or detergents to neutralize coexisting antibodies, such as hepatitis B surface antibody (HBsAb), and to release the antigen from immune complexes. Moreover, this pretreatment disrupts antigen polymers formed by hydrophobic bonding, thereby converting them into monomers and increasing the antigen's molecular count. Consequently, iTACT assays enable precise and highly sensitive measurement of the target antigen. Specifically, the detection thresholds of iTACT-HBsAg and iTACT-HBcrAg are 0.0005 IU/mL and 2.1 log U/mL, respectively. These assays are approximately 10 times more sensitive than established methods, including the HBsAg-HQ assay (detection threshold: 0.005 IU/mL) and conventional HBcrAg assay (detection threshold: 3.0 logU/mL).

In the clinical setting, Suzuki et al. reported that iTACT-HBcrAg and iTACT-HBsAg could predict HCC development in patients having achieved HBsAg clearance.^7^ Hosaka et al. provided real-world evidence that iTACT-HBcrAg was superior to conventional HBcrAg in prognosticating HCC during antiviral therapy in patients treated with entecavir.^8^ Indeed, iTACT-HBcrAg and iTACT-HBsAg are now emerging as viable tools to evaluate HBV activity in patients with HBsAg seroclearance, although more evidence is needed; for instance, the detection rate of iTACT-HBcrAg and iTACT-HBsAg in HBsAg seroclearance patients should be examined to identify high HCC risk from the perspective of HBV replication activity.

The present cross-sectional investigation examined the detection rate of iTACT-HBcrAg and iTACT-HBsAg in HBV patients with conventional assay-determined HBsAg seroclearance.

A total of 55 patients who had visited Shinshu University Hospital (Matsumoto, Japan) for HBV infection management between January 1, 2021, and December 31, 2021, were retrospectively targeted. Median age was 71 years, and 60.0% of subjects were male. The median period after HBsAg clearance was 5.1 years. Patients exhibiting other causes of chronic liver disease, including hepatitis C infection, alcoholic liver disease, nonalcoholic fatty liver disease, primary biliary cholangitis, and autoimmune hepatitis, were not considered. The racial background of all individuals was Japanese. Serum HBcrAg and HBsAg were measured by ultrasensitive assays based on iTACT technology (Fujirebio Inc., Tokyo, Japan) using patient serum samples immediately stored at −30 °C after collection. Samples obtained at the final visit in 2021 were used for this study.

**Figure 1:**
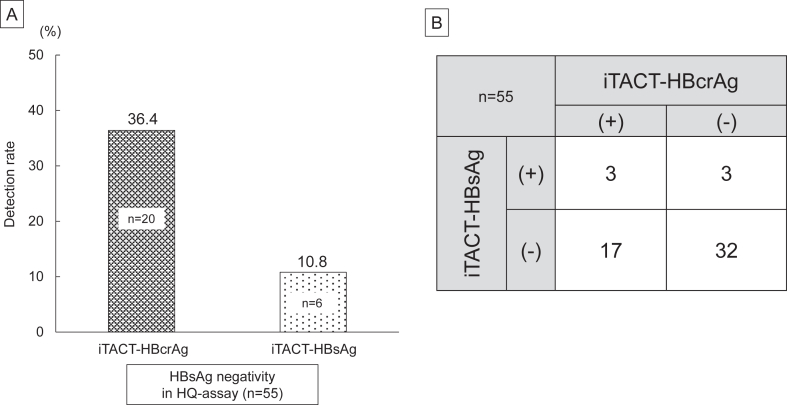
(A) Detection rates of HBcrAg and HBsAg by iTACT methods in patients with HBsAg-HQ negativity. (B) Contingency Table on iTACT-HBcrAg and iTACT-HBsAg results in patients with HBsAg-HQ negativity.

The detection rates of iTACT-HBcrAg and iTACT-HBsAg in the HQ-assay-determined HBsAg (−) group (n = 55) were examined first. Twenty cases (36.4%) and 6 cases (10.8%) were either HBcrAg (+) or HBsAg (+) according to the iTACT-HBcrAg method and iTACT-HBsAg method, respectively (Figure 1A). There were 3 cases in which both iTACT-HBcrAg and iTACT-HBsAg were positive (Figure 1B). The clinical characteristics of the iTACT-HBcrAg (−) and (+) groups are compared in Supplementary Table 1, showing no significant differences between the groups.

**Figure 2:**
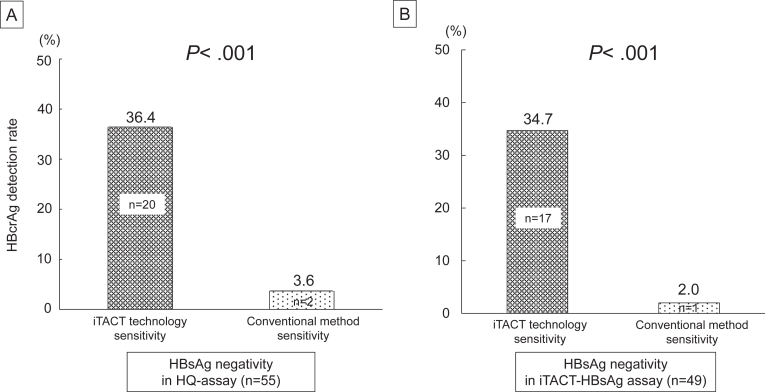
Comparison of the HBcrAg detection rates between iTACT-HBcrAg and the conventional HBcrAg method in the HBsAg-HQ (−) group (A) and the iTACT-HBsAg (−) group (B) (Chi-squared test).

Next, the detection rates of HBcrAg using the conventional sensitivity HBcrAg assay and the iTACT-HBcrAg assay were examined in the HBsAg (−) group as determined by the HQ-assay (n = 55) or iTACT-HBsAg (n = 49). In the HBsAg-HQ assay (−) group, 20 cases (36.4%) and 2 cases (3.6%) were HBcrAg (+) by iTACT-HBcrAg and the conventional HBcrAg method, respectively (Figure 2A). In the iTACT-HBsAg (−) group, 17 cases (34.7%) and 1 case (2.0%) were HBcrAg (+) by iTACT-HBcrAg and the conventional HBcrAg method, respectively (Figure 2B).

The detection rates of iTACT-HBcrAg and iTACT-HBsAg in patients with HCC history were examined last. Of all patients (n = 55), 7 patients had a history of HCC, 3 of whom tested positive for iTACT-HBcrAg, including one who also tested positive for iTACT-HBsAg. On the other hand, all 7 patients tested negative for conventional HBcrAg and HBsAg-HQ.

When considering our findings, iTACT-HBcrAg and iTACT-HBsAg detected HBcrAg in 20 cases (36.4%) and HBsAg in 6 cases (10.8%) in the HBsAg-HQ (−) group. Based on this result, iTACT-HBcrAg may have a higher detection rate than iTACT-HBsAg in seroclearance patients with HBsAg-HQ negativity at approximately 5 years. In addition, iTACT-HBcrAg showed a significantly higher detection rate of HBcrAg in patients with HBsAg seroclearance than the conventional HBcrAg method (Figure 2A). Similar results were observed even in patients with iTACT-HBsAg negativity (Figure 2B). Thus, the iTACT-HBcrAg method may enhance the detection of patients with potential HBV replication activity who cannot be identified by conventional means.

This study demonstrated the efficacy of iTACT-HBcrAg measurement in discerning potential HBcrAg positivity after HBsAg clearance. We witnessed no significant differences in clinical characteristics between the iTACT-HBcrAg (−) and (+) groups. However, our findings showed that 36% of HBsAg (−) cases in the HQ-assay were iTACT-HBcrAg (+) at a median of 5.1 years after achieving HBsAg seroclearance, suggesting that clinicians pay careful attention to carcinogenesis and HBV reactivation even after functional cure.

In conclusion, this investigation revealed the detection sensitivity of the iTACT-HBcrAg and iTACT-HBsAg methods in HBV patients with HBsAg seroclearance status. Further longitudinal large-scale studies are needed to confirm their clinical usefulness.
