# Supplementary material for: Detection Rates of Hepatitis B Surface and Core-related Antigens Using Novel Highly Sensitive Assays in Chronic Hepatitis B Patients With Hepatitis B Surface Antigen Seroclearance
Source: Gastro Hep Adv. 2024 Jul 3;3(7):885–7. doi: 10.1016/j.gastha.2024.06.013 (PMC11402281; doi:10.1016/j.gastha.2024.06.013)
Supplement: Supplementary Table 1 [file mmc1.docx]

**Supplementary Table 1: Characteristics of HBsAg-negative patients**

|  | All patients  (n=55) | iTACT-HBcrAg (-)  (n=35) | iTACT-HBcrAg (+)  (n=20) | *P*-value |
| --- | --- | --- | --- | --- |
|  | Median (IQR) |  |  |  |
| Age, years | 71 (61–77) | 73 (62–78) | 70 (60–72) | .532 |
| Male, n (%) | 33 (60.0%) | 20 (57.1%) | 13 (65.0%) | .567 |
| PLT, x10^4^/µL | 20.0 (13.8–23.9) | 17.8 (13.0–24.5) | 20.3 (15.0–22.2) | .506 |
| Alb, g/dL | 4.3 (4.0–4.5) | 4.3 (4.1–4.5) | 4.3 (4.0–4.5) | .535 |
| T. Bil, mg/dL | 0.87 (0.64–1.11) | 0.83 (0.64–1.02) | 0.90 (0.68–1.23) | .095 |
| AST, U/L | 22 (18–27) | 22 (18–26) | 22 (20–30) | .860 |
| ALT, U/L | 16 (13–22) | 15 (13–22) | 16 (13–21) | .546 |
| iTACT-HBcrAg, log U/mL | N/D (N/D–2.3) | N/D | 2.5 (2.2–2.7) | <.001 |
| HBV DNA, log IU/mL | N/D | N/D | N/D | 1.000 |
| HCC history, n (%) | 6 (10.9%) | 3 (8.6%) | 3 (15.0%) | .658 |
| Period after HBsAg clearance, years | 5.1 (1.7–7.1) | 5.0 (1.6–7.9) | 5.4 (2.1–6.7) | .913 |

**Abbreviations:** HBsAg, hepatitis B surface antigen; HBcrAg, hepatitis B core-related antigen; IQR, interquartile range; PLT, platelet count; Alb, albumin; T. Bil, total bilirubin; AST, aspartate aminotransferase; ALT, alanine aminotransferase; HBV, hepatitis B virus; HCC, hepatocellular carcinoma; N/D, not detected.
