# Supplementary material for: Detection Rates of Hepatitis B Surface and Core-related Antigens Using Novel Highly Sensitive Assays in Chronic Hepatitis B Patients With Hepatitis B Surface Antigen Seroclearance
Source: Gastro Hep Adv. 2024 Jul 3;3(7):885–7. doi: 10.1016/j.gastha.2024.06.013 (PMC11402281; doi:10.1016/j.gastha.2024.06.013)
Supplement: Supplemental Text [file mmc2.docx]

**Ethics**

This investigation was reviewed and approved by the Institutional Review Board of Shinshu University School of Medicine (approval number: 3244). All researchers involved in this study adhered to the tenets outlined in the Declaration of Helsinki (revised in 2013 by Fortaleza) and the Ethical Guidelines for Medical Research Involving Human Subjects (partially revised on February 28, 2017). An opt-out system is in place at our institution, whereby all information on the protocol and conduct of the study, including its purpose, is available on the Department of Medicine, Shinshu University School of Medicine website (http://www.shinshu-u.ac.jp/faculty/medicine/chair/i-2nai/). If patients do not wish to participate in the research, they are freely able to opt out of the study.

**Statistical analysis**

Statistical analysis and data visualization were carried out using StatFlex software ver. 7.0.11 (Artech Co., Ltd., Osaka, Japan). For clinical data analysis, groups were compared by means of the chi-squared test for categorical variables. Continuous baseline data are expressed as the median ± interquartile range. All statistical tests were two-sided and evaluated at the 0.05 level of significance.
